# The value of red blood cell distribution width in perioperative cardiac function assessment in children with congenital heart disease: A retrospective cohort study

**DOI:** 10.1097/MD.0000000000041144

**Published:** 2024-12-27

**Authors:** Qingsong Wang, Jun Yin, Xianmin Wang, Tongyong Luo, Min Wei, Xiaomeng Zhang, Huimin Ou, Weiyi Wan, Fuyan Li, Yundong Zhang, Caiyu Guo

**Affiliations:** a Department of Pediatrics, The First People’s Hospital of Jintang County/Jintang Hospital, West China Hospital, Sichuan University, Chengdu, Sichuan, China; b Department of Ultrasound, The First People’s Hospital of Jintang County/Jintang Hospital, West China Hospital, Sichuan University, Chengdu, Sichuan, China; c Pediatric Cardiology Center, Sichuan Provincial Women’s and Children’s Hospital/The Affiliated Women’s and Children’s Hospital of Chengdu Medical College, Chengdu, Sichuan, China; d Department of Epidemiology and Statistics, School of Public Health, Chengdu Medical College, Chengdu, Sichuan, China.

**Keywords:** cardiac function, congenital heart disease (CHD), perioperative, red blood cell distribution width (RDW)

## Abstract

Congenital heart disease (CHD) is the leading cause of birth defects in children, with high morbidity and mortality rates. Despite advances in surgical and interventional treatments, perioperative management of CHD remains challenging, particularly in complex cases and neonates. Effective perioperative assessment of cardiac function is essential for optimizing surgical outcomes and improving postoperative prognosis. This study investigates the association between red blood cell distribution width (RDW) and perioperative cardiac function in children with CHD. Specifically, we aim to determine whether RDW can serve as a reliable biomarker for assessing perioperative cardiac risk in this patient population. This retrospective cohort study included 107 pediatric patients aged 0 to 18 years who underwent surgical or interventional treatment for CHD at Sichuan Provincial Maternity and Child Health Hospital between March 2022 and March 2023. Patients were classified into 3 groups based on their cardiac function (Class I–III) according to the European Society of Cardiology guidelines. Clinical and imaging data, as well as RDW levels, were analyzed to assess their relationship with perioperative cardiac function. Both preoperative and postoperative RDW-coefficient of variation (RDW-CV) and RDW-standard deviation (RDW-SD) levels were significantly higher with increasing severity of cardiac dysfunction, with statistically significant differences observed among the 3 cardiac function groups (*P* < .001). Preoperative RDW-CV values were 13.26 ± 1.44%, 15.07 ± 2.13%, and 19.87 ± 3.62%, and RDW-SD values were 39.84 ± 4.04%, 45.02 ± 6.33%, and 56.12 ± 9.61% (all *P* < .001). Postoperative RDW-CV values were 14.05 ± 2.14%, 15.98 ± 2.29%, and 20.36 ± 4.42%, and RDW-SD values were 43.99 ± 4.73%, 48.21 ± 6.23%, and 58.33 ± 9.71% (all *P* < .001). Patients with heart failure had significantly higher RDW-CV and RDW-SD levels compared to non-heart failure patients, both preoperatively and postoperatively (*P* < .001). RDW is strongly associated with perioperative cardiac function in children with CHD. RDW levels increase with the severity of cardiac dysfunction and are clinically valuable in identifying heart failure in these children. As a simple and effective biomarker, RDW can assist in the perioperative assessment of cardiac function in this patient population.

## 1. Introduction

Congenital heart disease (CHD) is the most common congenital malformation among children in China, with an overall prevalence of 0.8% to 1.2%, and it accounts for the highest incidence and mortality rate among major birth defects.^[1,2]^ Although surgical and interventional treatment techniques for CHD have become well-established, surgery for complex and neonatal CHD patients still presents numerous challenges and risks.^[3,4]^ Particularly in the perioperative period for these high-risk patients, accurate assessment of cardiac function is critical for the success of the surgery and for postoperative prognosis.

Red blood cell distribution width (RDW) is an index that measures the variation in red blood cell volume, initially used for the differential diagnosis of anemia. As a routine and easily accessible hematological marker, RDW has gradually been applied in the risk assessment and prognosis prediction of various diseases. Recent studies have shown that RDW is closely associated with cardiovascular diseases, such as arrhythmias, heart failure, and coronary artery disease.^[5]^ It is considered a significant predictor for both acute and chronic cardiovascular conditions and is closely related to the prognosis of cardiovascular surgeries and interventional treatments. Current research^[6–8]^ indicates that RDW is strongly correlated with surgical complications and postoperative outcomes in CHD patients. Therefore, further investigation into the relationship between RDW and cardiac function in CHD children during the perioperative period is of great importance. In addition, monitoring changes in RDW levels may help explore the physiological and pathological mechanisms underlying cardiac dysfunction, providing a more comprehensive understanding of the risks associated with other cardiovascular diseases.

This study aims to analyze the changes in RDW levels during the perioperative period of CHD and investigate its relationship with cardiac function in CHD children. The goal is to offer a new diagnostic marker for perioperative cardiac function assessment and provide robust evidence for clinical decision-making.

## 2. Materials and methods

### 2.1. Study population

This retrospective study included 107 pediatric patients, aged 0 to 18 years, who underwent surgical or interventional treatment for CHD at Sichuan Provincial Maternity and Child Health Hospital between March 2022 and March 2023. The inclusion criteria were as follows: (1) children aged 0 to 18 years with a diagnosis of CHD; (2) patients who met the surgical indications for CHD correction; (3) those with cardiac function classified as Class I to III according to the European Society of Cardiology guidelines; and (4) patients with complete clinical and imaging data. The exclusion criteria included: (1) children with CHD complicated by trisomy 21 or genetic metabolic disorders; and (2) patients with a history of previous cardiac surgery.

### 2.2. Data collection

① General information: including age, gender, and type of CHD.
② Preoperative data: complete blood count (RDW-coefficient of variation [RDW-CV] and RDW-standard deviation [RDW-SD]), biochemical markers (lactate dehydrogenase [LDH], α-hydroxybutyrate dehydrogenase [α-HBDH]), cardiac biomarkers (creatine kinase [CK], creatine kinase MB isoenzyme [CK-MB], B-type natriuretic peptide [BNP], cardiac troponin I [cTnI]), echocardiography, and left ventricular ejection fraction (LVEF).
③ Postoperative data: complete blood count (RDW-CV, RDW-SD), biochemical markers (LDH, α-HBDH), cardiac biomarkers (CK, CK-MB, BNP, cTnI), echocardiography, and LVEF.

### 2.3. Cardiac function classification and heart failure diagnosis

Cardiac function was classified and heart failure diagnosed according to the European Society of Cardiology guidelines. Based on cardiac function, patients were divided into 3 groups: Class I (n = 51), Class II (n = 32), and Class III (n = 24). Since Class IV cases were rare, some Class III to IV cases were grouped into Class III. Additionally, patients were classified into 2 groups based on heart failure status: heart failure group (n = 26) and non-heart failure group (n = 81).

### 2.4. Blood tests and biochemical analysis

Venous blood samples were collected from all CHD children in the morning after hospital admission and on the first postoperative day, following an overnight fast. RDW levels (including RDW-CV and RDW-SD) were measured using the SYSMES XN-9000 hematology analyzer. Biochemical markers and cardiac biomarkers, including BNP, were analyzed using the COBAS C702 automated biochemical analyzer.

### 2.5. Echocardiography

Echocardiographic examinations were performed using Doppler ultrasound to assess cardiac function and record LVEF values. These were measured within 1 month preoperatively and on the first postoperative day.

### 2.6. Statistical analysis

Data analysis was performed using SPSS 26.0 software. Normality was first tested; for normally distributed measurement data, *t* tests were used for comparisons between 2 groups, and chi-square tests were used for categorical data. ANOVA was used for comparisons among multiple groups. Non-normally distributed data were analyzed using the nonparametric rank-sum test. The correlation between RDW-CV and RDW-SD was assessed using Pearson correlation analysis. An ordinal logistic regression model was used to analyze the influencing factors of preoperative cardiac function classification, and binary logistic regression was applied to predict the occurrence of preoperative heart failure. A *P* value of <.05 was considered statistically significant.

## 3. Results

### 3.1. General information comparison

A total of 107 children undergoing perioperative surgical or interventional treatment for CHD were included in this study. The distribution of cardiac function classifications was as follows: Class I (n = 51, 47.66%), Class II (n = 32, 29.91%), and Class III (n = 24, 22.43%). There were 44 male and 63 female patients, with no significant difference in gender distribution among the 3 groups (*P* = .92). The median ages (M, P25–P75) were 2.64 years (1–5 years) in Class I, 2.11 years (0.55–4 years) in Class II, and 0.90 years (0.33–2.75 years) in Class III, with no statistically significant difference in age among the groups (*P* = .228). The proportion of patients with heart failure increased significantly with worsening cardiac function (*P* < .05), as shown in Table 1.

**Table 1:** Comparison of general information on cardiac function among 3 groups.

Cardiac function grouping	Class I (n = 51)	Class II (n = 32)	Class III (n = 24)	Total	*F/χ2*	*P* value
HF (%)	2 (0.039)	5 (0.156)	19 (79.17)	26	0.471	<.001
Gender (cases) male	20	14	10	44	0.386	.92
Female	31	18	14	63		
Age [years, median (P25–P75)]	2.64 (1–5)	2.1 (0.55–4)	0.9 (0.33–2.75)		0.259	.228

HF = heart failure.

### 3.2. Comparison of preoperative and postoperative RDW

#### 3.2.1. Preoperative comparison among cardiac function groups

The levels of CK, CK-MB, LVEF, LDH, α-HBDH, RDW-CV, RDW-SD, BNP, and cTnI were compared among the cardiac function groups I, II, and III before CHD surgery. There were no statistically significant differences in preoperative CK-MB and LVEF levels (F = 4.29, 2.847, 1.919; all *P* > .05). However, significant differences were observed in preoperative CK, LDH, α-HBDH, RDW-CV, RDW-SD, BNP, and cTnI levels (F = 4.29, 14.042, 11.52, 68.24, 53.691, 8.807, 19.216; all *P* < .001), as shown in Table 2.

**Table 2:** Comparison of various indicators among patients with 3 grades of preoperative cardiac function.

Cardiac function grouping	Class I	Class II	Class III	F value	*P* value
Preoperative CK	136.76 ± 74.271	89.19 ± 59.873	164.13 ± 164.696	4.29	.016
Preoperative LDH	271.843 ± 66.5866	304.391 ± 148.1224	419.917 ± 138.554	14.042	<.001
Preoperative α-HBDH	216.73 ± 58.976	249.13 ± 115.141	331.13 ± 128.707	11.52	<.001
Preoperative RDW-CV	13.255 ± 1.4413	15.066 ± 2.1249	19.867 ± 3.6194	68.254	<.001
Preoperative RDW-SD	39.843 ± 4.0425	45.022 ± 6.3331	56.117 ± 9.613	53.691	<.001
Preoperative CK-MB	4.0304 ± 2.0800	3.0719 ± 1.79002	3.3161 ± 1.5060	2.847	.063
Preoperative BNP	283.43 ± 398.37	750.66 ± 1577.57	4960.04 ± 9696.94	8.807	<.001
Preoperative cTnI	11.376 ± 9.0768	20.159 ± 26.9013	92.871 ± 111.297	19.216	<.001
Preoperative LVEF	66.373 ± 6.3181	62.747 ± 13.0522	62.583 ± 10.656	1.919	.152

BNP = B-type natriuretic peptide, CK = creatine kinase, CK-MB = creatine kinase MB isoenzyme, cTnI = cardiac troponin I, LDH = lactate dehydrogenase, LVEF = left ventricular ejection fraction, RDW-CV = red cell volume distribution width-coefficient of variation, RDW-SD = red cell distribution width-standard deviation, α-HBDH = α-hydroxybutyrate dehydrogenase.

#### 3.2.2. Postoperative comparison among cardiac function groups

There were no statistically significant differences in postoperative CK, LDH, and LVEF levels (F = 3.256, 3.566, 2.167; all *P* > .05). However, significant differences were observed in postoperative α-HBDH, RDW-CV, RDW-SD, CK-MB, BNP, and cTnI levels among the groups (F = 7.106, 40.11, 38.95, 8.632, 9.638, 7.451; all *P* < .05), as shown in Table 3.

**Table 3:** Comparison of various indices among patients with different postoperative cardiac function classifications.

Cardiac Function Grouping	Class I	Class II	Class III	F value	*P* value
Postoperative CK	866.51 ± 670.718	585.38 ± 383.4221	938.79 ± 569.946	3.256	.053
Postoperative LDH	463.63 ± 240.025	569.38 ± 673.526	764.88 ± 451.324	3.566	.052
Postoperative α-HBDH	371.98 ± 201.879	406.72 ± 309.209	622.83 ± 352.279	7.106	.001
Postoperative RDW-CV	14.049 ± 2.1439	15.981 ± 2.287	20.363 ± 4.4169	40.11	<.001
Postoperative RDW-SD	43.986 ± 4.7267	48.209 ± 6.2341	58.333 ± 9.7048	38.95	<.001
Postoperative CK-MB	11.909 ± 9.191	15.353 ± 12.830	23.836 ± 14.259	8.632	<.001
Postoperative BNP	3436.48 ± 4518.17	6095.0 ± 5401.231	9445.46 ± 7463.66	9.638	<.001
Postoperative ctni	406.114 ± 455.744	1229.2 ± 1584.493	999.938 ± 798.789	7.451	.001
Postoperative LVEF	62.14 ± 6.94	58.5 ± 10.698	58.92 ± 8.757	2.167	.120

BNP = B-type natriuretic peptide, CK = creatine kinase, CK-MB = creatine kinase MB isoenzyme, cTnI = cardiac troponin I, LDH = lactate dehydrogenase, LVEF = left ventricular ejection fraction, RDW-CV = red cell volume distribution width-coefficient of variation, RDW-SD = red cell distribution width-standard deviation, α-HBDH = α-hydroxybutyrate dehydrogenase.

#### 3.2.3. Comparison between heart failure and non-heart failure groups preoperatively

The preoperative levels of CK, CK-MB, LVEF, LDH, α-HBDH, RDW-CV, RDW-SD, BNP, and cTnI were compared between the heart failure and non-heart failure groups. No statistically significant differences were found in preoperative CK and CK-MB levels (t = 1.134, 0.633; both *P* > .05). However, significant differences were observed in LDH, α-HBDH, RDW-CV, RDW-SD, BNP, cTnI, and LVEF levels between the groups (t = 4.744, 3.889, 7.612, 8.922, 2.445, 5.443, 2.325; all *P* < .05), as shown in Table 4.

**Table 4:** Comparison of various indices between patients with and without heart failure before surgery.

	Heart failure	Non-heart failure	t value	*P* value
Preoperative CK	148.31 ± 164.216	122.37 ± 71.308	1.134	.259
Preoperative LDH	408.423 ± 149.7721	284.735 ± 102.7272	4.744	<.001
Preoperative α-HBDH	317.85 ± 125.258	230.96 ± 89.377	3.889	<.001
Preoperative RDW-CV	18.892 ± 3.0326	14.12 ± 2.6982	7.612	<.001
Preoperative RDW-SD	55.377 ± 9.1365	41.725 ± 5.8657	8.922	<.001
Preoperative CK-MB	3.3732 ± 1.50592	3.6517 ± 2.03058	-0.633	.528
Preoperative BNP	3502.19 ± 7896.278	820.49 ± 3406.169	2.445	.016
Preoperative cTnI	84.562 ± 105.9532	15.501 ± 25.5019	5.443	<.001
Preoperative LVEF	60.615 ± 10.8815	65.665 ± 9.2139	-2.325	.022

BNP = B-type natriuretic peptide, CK = creatine kinase, CK-MB = creatine kinase MB isoenzyme, cTnI = cardiac troponin I, LDH = lactate dehydrogenase, LVEF = left ventricular ejection fraction, RDW-CV = red cell volume distribution width-coefficient of variation, RDW-SD = red cell distribution width-standard deviation, α-HBDH = α-hydroxybutyrate dehydrogenase.

#### 3.2.4. Comparison between heart failure and non-heart failure groups postoperatively

There were no statistically significant differences in postoperative CK, LDH, cTnI, and LVEF levels (t = 1.455, 1.459, 0.620, −1.444; all *P* > .05). However, significant differences were found in postoperative α-HBDH, RDW-CV, RDW-SD, CK-MB, and BNP levels (t = 3.377, 5.440, 6.083, 3.598, 3.817; all *P* < .05), as shown in Table 5.

**Table 5:** Comparison of various indicators among patients with 3 grades of postoperative cardiac function.

	Heart failure	Non-heart failure	t value	*P* value
Postoperative CK	943.962 ± 534.373	752.002 ± 600.202	1.455	.149
Postoperative LDH	678.46 ± 415.156	525.7 ± 478.842	1.459	.148
Postoperative α-HBDH	598.23 ± 284.677	387.41 ± 274.514	3.377	.001
Postoperative RDW-CV	19.135 ± 3.5957	15.039 ± 3.2486	5.440	<.001
Postoperative RDW-SD	56.185 ± 8.5021	45.99 ± 7.069	6.083	<.001
Postoperative CK-MB	22.8381 ± 13.46631	13.2958 ± 11.18139	3.598	<.001
Postoperative BNP	9263.38 ± 7129.888	4387.49 ± 5085.554	3.817	<.001
Postoperative ctni	897.265 ± 810.599	749.553 ± 1122.362	0.620	.536
Postoperative LVEF	58.19 ± 8.025	61.01 ± 8.856	-1.444	.152

BNP = B-type natriuretic peptide, CK = creatine kinase, CK-MB = creatine kinase MB isoenzyme, cTnI = cardiac troponin I, LDH = lactate dehydrogenase, LVEF = left ventricular ejection fraction, RDW-CV = red cell volume distribution width-coefficient of variation, RDW-SD = red cell distribution width-standard deviation, α-HBDH = α-hydroxybutyrate dehydrogenase.

### 3.3. Correlation analysis between RDW-CV and RDW-SD

In children with CHD, preoperative RDW-CV was significantly correlated with preoperative RDW-SD, postoperative RDW-CV, and postoperative RDW-SD (*R* = 0.788, 0.863, 0.807; all *P* < .001), as shown in Table 6.

**Table 6:** Correlation analysis between RDW-CV and RDW-SD.

		Preoperative RDW-CV	Preoperative RDW-SD	Postoperative RDW-CV	Postoperative RDW-SD
Preoperative RDW-CV	Pearson correlation	1	0.788	0.863	0.807
	Sig.		<0.001	<0.001	<0.001
Preoperative RDW-SD	Pearson correlation		1	0.631	0.776
	Sig.			<0.001	<0.001
Postoperative RDW-CV	Pearson correlation			1	0.894
	Sig.				<0.001
Postoperative RDW-SD	Pearson correlation				1

RDW-CV = red cell volume distribution width-coefficient of variation, RDW-SD = red cell distribution width-standard deviation.

### 3.4. Ordered logistic regression analysis of preoperative cardiac function

Preoperative RDW-CV was significantly positively correlated with cardiac function classification (B = 0.508, *P* = .003), indicating that an increase in preoperative RDW-CV is closely related to worsening cardiac function, as shown in Table 7.

**Table 7:** Ordered logistic regression test for cardiac function in 3 groups before congenital heart disease surgery.

	B	SE	Wald	F value	95% CI low	95% CI high
Preoperative LDH	-0.006	0.006	0.94	0.332	-0.019	0.006
Preoperative α-HBDH	0.009	0.008	1.292	0.256	-0.006	0.024
Preoperative RDW-CV	0.486	0.164	8.821	0.003	0.165	0.807
Preoperative RDW-SD	0.081	0.057	1.968	0.161	-0.032	0.193
Preoperative BNP	0	0	1.754	0.185	0	0.001
Preoperative cTnI	0.015	0.009	2.631	0.105	-0.003	0.034

BNP = B-type natriuretic peptide, cTnI = cardiac troponin I, HF = heart failure, LDH = lactate dehydrogenase, RDW-CV = red cell volume distribution width-coefficient of variation, RDW-SD = red cell distribution width-standard deviation, α-HBDH = α-hydroxybutyrate dehydrogenase.

### 3.5. Logistic regression analysis of heart failure

Preoperative RDW-SD was significantly associated with the occurrence of heart failure (*P* < .05), suggesting that preoperative RDW-SD may be an important biomarker for predicting preoperative heart failure in children with CHD, as shown in Table 8.

**Table 8:** Logistic regression analysis of heart failure occurrence before congenital heart disease surgery.

	B	SE	Wald	F value	Exp (B)
Preoperative LDH	-0.001	0.016	0.002	0.965	0.999
Preoperative α-HBDH	0.000	0.019	0.000	0.988	10.000
Preoperative RDW-CV	-0.272	0.297	0.840	0.360	0.762
Preoperative RDW-SD	0.302	0.128	50.547	0.019	10.352
Preoperative BNP	0.000	0.001	0.311	0.577	10.000
Preoperative cTnI	0.033	0.021	20.493	0.114	10.034
Preoperative LVEF	-0.018	0.034	0.276	0.599	0.982

BNP = B-type natriuretic peptide, cTnI = cardiac troponin I, LDH = lactate dehydrogenase, LVEF = left ventricular ejection fraction, RDW-CV = red cell volume distribution width-coefficient of variation, RDW-SD = red cell distribution width-standard deviation, α-HBDH = α-hydroxybutyrate dehydrogenase.

## 4. Discussion

CHD is among the most common congenital anomalies in children. Although advancements in cardiac surgery and interventional techniques have significantly improved outcomes, the perioperative period still poses high risks, especially for children with compromised cardiac function. Therefore, accurate assessment of cardiac function in CHD patients is crucial for improving survival rates and quality of life. Previous studies, such as those by Liu Sen et al,^[9]^ have shown that RDW is associated with CHD, particularly in children with heart failure. Elevated RDW levels have been linked to poor postoperative recovery and increased in-hospital mortality in CHD patients.^[10]^ Despite RDW’s growing use in predicting complications and disease severity in CHD, limited research has focused on its relationship with cardiac function during the perioperative period in pediatric patients.

In this study, we found that younger children with more complex forms of CHD were more likely to have associated vascular abnormalities and require more intensive surgical or interventional treatment. These findings align with those of Li Gang et al^[10]^ and Li Kangming et al,^[11]^ who emphasized the benefits of early surgical intervention in improving survival, particularly in infants and neonates with severe CHD. Notably, our study showed that the incidence of heart failure increased as cardiac function worsened, underscoring the close relationship between cardiac dysfunction and the development of heart failure in children with CHD.

Our analysis also demonstrated a significant correlation between both RDW-CV and RDW-SD with cardiac function. RDW levels increased as cardiac function declined, both preoperatively and postoperatively, suggesting that RDW could serve as an indicator of the severity of cardiac dysfunction in CHD patients during the perioperative period. This finding is consistent with those of Lu Yahang et al^[12]^ and Nagaoka Kota et al,^[13]^ who reported a strong association between RDW and disease severity in pediatric CHD patients. Other studies^[6–8,14,15]^ have similarly linked elevated preoperative RDW levels to poor outcomes and complications in critical CHD cases, including prolonged mechanical ventilation and extended hospital stays. These associations highlight the potential utility of RDW in the early identification and postoperative monitoring of critically ill CHD patients.

Furthermore, our study revealed that RDW-SD and BNP levels were significantly higher in the heart failure group compared to the non-heart failure group, suggesting that both RDW and BNP are valuable in diagnosing heart failure in children with CHD. Fukuta H. et al^[16]^ were among the first to report a strong correlation between RDW and BNP in cardiovascular disease. BNP, a well-established biomarker, reflects cardiac function not only in left ventricular systolic dysfunction but also in diastolic dysfunction, valvular disease, and right ventricular dysfunction. Neurohumoral regulation of the heart may influence RDW levels.^[17]^ Additionally, Hong Ping et al^[18]^ and Li J et al^[19]^ observed that RDW levels increased with the severity of both acute and chronic heart failure in CHD patients, correlating with changes in N-terminal pro-B-type natriuretic peptide levels and LVEF. These findings further support the role of RDW in assessing heart failure severity in CHD patients.

Interestingly, our logistic regression analysis found that BNP was not significantly associated with heart failure when compared to RDW-SD. This may be because CHD patients had already received treatments such as inotropes and diuretics, potentially altering BNP levels and affecting the analysis. This result suggests that, in clinical practice, multiple factors should be considered when evaluating the cardiac function of CHD patients.

Several studies^[20–22]^ have identified cardiac metabolic markers, such as CK, CK-MB, LDH, and α-HBDH, as important indicators of myocardial injury and useful reference markers for assessing cardiac function. Our study confirmed that preoperative levels of LDH, α-HBDH, RDW-CV, and RDW-SD increased with worsening cardiac function. Similarly, postoperative levels of CK-MB, α-HBDH, RDW-CV, and RDW-SD showed a parallel trend. Notably, children with CHD and poor cardiac function exhibited even higher levels of α-HBDH and CK-MB post-surgery, indicating the impact of CHD surgery and interventional therapy on the myocardium. Chen Huiying et al^[23]^ also reported that BNP, CK-MB, CK, LDH, and α-HBDH levels in the serum of CHD patients are closely related to disease severity and can effectively reflect cardiac function, which is consistent with our findings. These results suggest that RDW, in combination with other cardiac metabolic markers, could be used to assess cardiac function, heart failure severity, and myocardial injury, thereby guiding surgical decisions and improving postoperative outcomes.

Although this study provides an in-depth investigation into the relationship between RDW and perioperative cardiac function in children with CHD, it does not address the impact of RDW level changes on postoperative quality of life or functional status. The assessment of quality of life and functional status is crucial for understanding the comprehensive recovery of patients after surgery, especially in high-risk pediatric populations. As clinical treatment methods continue to advance, focusing on the overall health status and functional recovery of patients, particularly the evaluation of postoperative quality of life, holds significant clinical importance.

Future studies should consider utilizing specific tools to assess the relationship between RDW level changes and postoperative quality of life and functional status in children with CHD. For example, standardized quality of life questionnaires, such as the SF-36, or functional assessment scales, could be used to further explore the connection between RDW and postoperative recovery. This approach will contribute to a more comprehensive evaluation of the potential role of RDW in postoperative prognosis in CHD patients, providing a more holistic basis for clinical decision-making.

One limitation of this study is the lack of comprehensive consideration of other potential factors that may influence RDW and cardiac function. For instance, variables such as the patient’s age, underlying health conditions, medications, and other clinical interventions could have an impact on RDW levels and cardiac function. Additionally, other physiological and pathological factors, such as systemic inflammatory responses or nutritional status, may also contribute to changes in RDW and postoperative cardiac function. As this study did not fully control for or analyze these factors, the potential influence of these variables on the results cannot be excluded. Future research should aim to incorporate these variables and employ more refined study designs to control for such confounding factors in order to obtain more accurate and reliable conclusions.

## 5. Conclusion

RDW is strongly associated with perioperative cardiac function in children with CHD. RDW levels increase with the severity of cardiac dysfunction and are clinically valuable in identifying heart failure in these children. As a simple and effective biomarker, RDW can assist in the perioperative assessment of cardiac function in this patient population.

## Acknowledgments

We acknowledge all the authors in this article.

## Author contributions

**Conceptualization:** Jun Yin, Xiaomeng Zhang.

**Data curation:** Qingsong Wang, Min Wei, Huimin Ou.

**Formal analysis:** Qingsong Wang, Min Wei.

**Investigation:** Xiaomeng Zhang, Caiyu Guo.

**Methodology:** Jun Yin, Xiaomeng Zhang, Weiyi Wan, Fuyan Li, Yundong Zhang, Caiyu Guo.

**Project administration:** Jun Yin, Yundong Zhang.

**Resources:** Xianmin Wang.

**Supervision:** Tongyong Luo.

**Validation:** Tongyong Luo, Huimin Ou, Fuyan Li.

**Writing – original draft:** Qingsong Wang.

**Writing – review & editing:** Xianmin Wang.
